# Synchronous bilateral tonsil carcinoma: case presentation and review of the literature

**DOI:** 10.1186/s13027-017-0146-5

**Published:** 2017-06-26

**Authors:** M-N. Theodoraki, J. A. Veit, T. K. Hoffmann, J. Greve

**Affiliations:** Department of Oto-Rhino-Laryngology, Head and Neck Surgery, University Medical Center, Frauensteige 12, 89070 Ulm, Germany

**Keywords:** Bilateral tonsillar carcinoma, Cancer of unknown primary, Head and neck malignancy, Squamous cell carcinoma, Bilateral tonsillectomy

## Abstract

**Background:**

The incidence of synchronous bilateral tonsil carcinoma seems to be underreported. For adequate oncologic treatment, it is mandatory to remove all primaries to prevent recurrence or metachronic disease. The purpose of this manuscript is to provide a comprehensive review on this topic and to emphasize the need of bilateral tonsillectomy in cases of cancer of unknown primary (CUP) as well as in the case of a unilateral tonsillar carcinoma.

**Material and methods:**

A systematic review of the literature was performed for “bilateral tonsillar neoplasm”, “synchronous cancer of the oropharynx” and “cancer of unknown primary in head and neck”.

**Results:**

We present a clinical case with bilateral tonsillar carcinoma in initially suggested cancer of unknown primary. Clinically, both tonsillar sites were unsuspicious, but in PET/CT an ipsilateral enhancement of the tonsil area was detected. The pathological work up of bilateral tonsillectomy specimens revealed bilateral squamous cell carcinoma with HPV-type 16 positivity. The review of the literature revealed 29 cases of bilateral tonsil cancer.

**Conclusion:**

The handling of tonsillar tissue in the frame of panendoscopy in the case of CUP is still controversial. We recommend a bilateral tonsillectomy as a routine procedure for cancer of unknown primary as well as unilateral tonsillar carcinoma. Herewith the detrimental consequences of occult metachronous contralateral tonsillar carcinoma can be prevented.

## Background

The detection and treatment of the primary neoplasm in cancer of unknown primary (CUP) of the head and neck (H&N) presents a challenge for the clinician. Currently, the recommended procedure involves imaging techniques as well as panendoscopy with systematic biopsies of localizations with high incidence of occult primary side including an ipsilateral tonsillectomy or at least a biopsy of the ipsilateral tonsil. At first sight, up to 10% of H&N malignancies present as a CUP [1] and the primary tumor can be identified in approximately 21, 5–75% of these cases. As in all H&N malignancies, squamous cell carcinoma presents the most common entity, with the tonsil being the most frequent localization for an occult primary. Patients with a CUP disease have a decreased overall survival rate compared to other head and neck squamous cell carcinoma (HNSCC) patients [2].

**Fig. 1:**
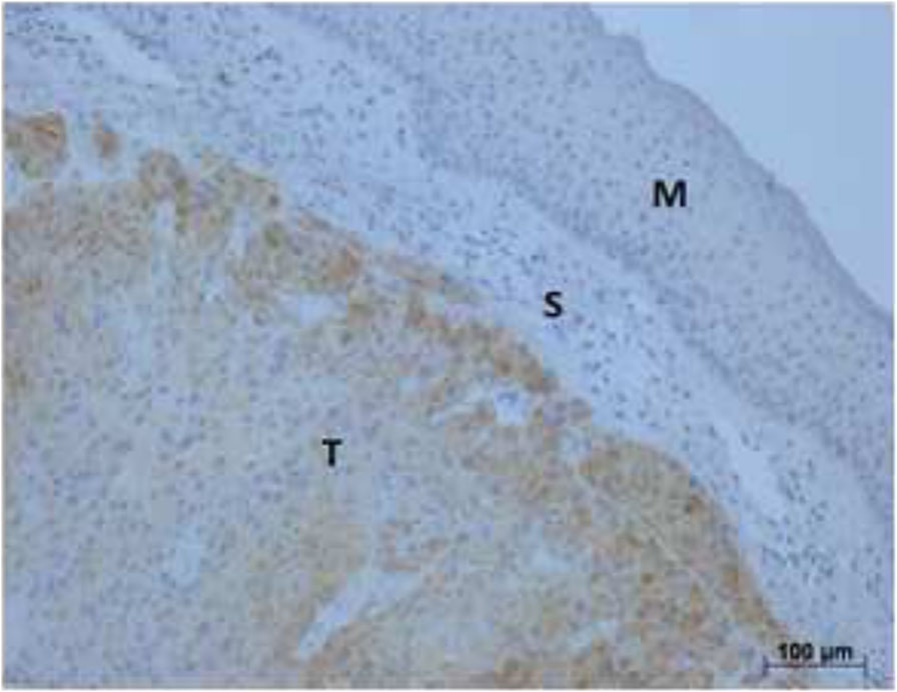
Immunhistochemical staining of cancer-testis-antigen MAGEA3/A4 with submucosal presentation of a tonsillar carcinoma. Figure of our unpublished data. T = tumor, S = stroma, M = mucosa

Primary tonsillar carcinoma is the third most common malignancy in H&N area, after thyroid and larynx carcinoma [3]. About 50% of patients are diagnosed with lymphatic metastazation, occurring on the contralateral cervical side in 10–15% [4]. Tonsillar malignancy is likely to be diagnosed in advanced stages with indolent neck mass due to frequent submucosal presentation or deep formations in the crypts with few local clinical symptoms (Fig. 1).

The presence of synchronous malignant tumors in the H&N is not uncommon and is the leading long-term cause of mortality [5]. Within five years, 15% of patients with a tonsillar carcinoma present a secondary tumor localized in the H&N [6]. The risk of a secondary malignant tumor in the H&N area is linked by the degree of symmetric chronic exposure to carcinogenic factors of the upper aerodigestive tract. In more recent investigations a strong association of oropharyngeal cancer and -in a lesser extend- CUP-syndromes with human papilloma viruses (HPV) is visible, with significantly better clinical outcome [7].

However, synchronous bilateral tonsil carcinoma is uncommon and only a few cases are reported in literature with the first report in 1971 [8]. The true incidence is likely to be underreported.

The high frequency of tonsillar primaries, as mentioned above, leads to frequent recommendation of unilateral diagnostic tonsillectomy in the context of CUP-panendoscopy [9]. If a tonsillar carcinoma is suspected, a panendoscopy with biopsy or tonsillectomy of the suspected (ipsilateral) tonsil follows. The recommendation for a bilateral tonsillectomy is frequently seen in literature [10] but without consistent performance in clinical practice or integration in corresponding guidelines.

We present a case of a synchronous bilateral tonsil carcinoma with subsequent review of the current literature. This article intends to raise the question of whether a bilateral tonsillectomy should be established as a standard procedure, with the aim of a homogenous approach in cases of cervical CUP-syndrome and/or unilateral tonsillar cancer.

## Main text

### Materials and methods

A systematic review of the literature was performed via MEDLINE using the terms “bilateral tonsillar neoplasm”, “cancer of unknown primary in head and neck” as well as “synchronous cancer of the oropharynx” from the years 1971 until 2016. All abstracts were reviewed and all publications mentioning a bilateral tonsillar carcinoma were included. The references in the relevant papers were also reviewed. We declare that we have read the Helsinki Declaration and have followed the guidelines in this investigation.

### Results

We identified 18 manuscripts describing 29 cases of synchronous bilateral tonsil carcinoma with one case presenting an additional contralateral carcinoma in situ and four cases of contralateral metachronous tonsillar carcinoma. The principal recommendations of these papers are shown in Table 1 [1, 3, 4, 7–21].

**Table 1:** Literature with previously reported cases of bilateral tonsillar carcinoma

Authors	Year	Country	synchr.	metachr.	Recommendation	HPV-16-status	Primary diagnosis
Patel	2015	USA	3	1	Cases in context of dysphagie after bilateral transoral resection	positive	3× CUP, 1× unilateral carcinoma
Bakkal	2014	Turkey	1		Case in context of primary chemoradiotherapy treatment	n.p.	Bilateral carcinoma
Nakahara	2014	Japan	1		Bilateral tonsillectomy or biopsy if HPV positivity	positive	CUP
Joseph	2014	USA	3	1	Bilateral tonsillectomy	positive	Unilateral carcinoma
Moualed	2011	UK	3		Bilateral tonsillectomy by suspected or proven tonsillar carcinoma	n.p.	Unilateral carcinoma
Mannina	2011	USA	1		Role of PET/CT staging for diagnosis of CUP	positive	CUP
Roeser (poster)	2011	USA	1		Bilateral tonsillectomy if bilateral tonsillar metastasation	positive	CUP
Smith (poster)	2011	USA	1	3	Bilateral tonsillectomy by CUP or unialteral tonsil carcinoma	n.p.	1× CUP, 3× unilateral carcinoma
Monsted	2010	Danemark	1		No recommendation in abstract, article in Danish	unknown	Unilateral carcinoma
Chianchetti	2009	USA	1		Unilateral (or less often) bilateral tonsillectomy by diagnosis of CUP	n.p.	CUP
McGovern	2009	USA	1		Bilateral tonsillectomy if both tonsills enlarged + positive PET-CT scan	positive	CUP
Kothari	2008	UK	5		Bilateral tonsillectomy by diagnosis of CUP	n.p.	CUP
Kozakiewicz	2007	Poland	1		Bilateral tonsillectomy by bilateral cervikal metastasation	unknown	Unilateral carcinoma
Price	2006	UK	1		Role of FDG-PET in diagnosis of CUP, search for primary side	n.p.	CUP
Kazak	2003	Germany	1		Bilateral tonsillectomy by diagnosis of CUP	n.p.	CUP
Koch	2001	USA	2^a^		Bilateral tonsillectomy by diagnosis of CUP	n.p.	CUP
Rajendekumar	1999	UK	1		Search for further head and neck primary	n.p.	Unilateral carcinoma
Schöndorf	1971	Germany		1	Bilateral tonsillectomy by diagnosis of unilateral tonsil carcinoma	n.p.	Unilateral carcinoma

*Synchr.* synchronous manifestation of bilateral tonsillar carcinoma, *metachr.* metachronous tonsillar carcinoma of the contralateral side; The numbers in the rows synchronous and metachronous demonstrate the number of reported cases in the according publications; ^a^1× contralateral carcinoma in situ; *n.p.* not performed

Furthermore, we describe a case of a bilateral tonsillar carcinoma confirmed by histopathological analysis following bilateral tonsillectomy in the context of panendoscopy for diagnosis of an occult primary.

#### Case

**Fig. 2:**
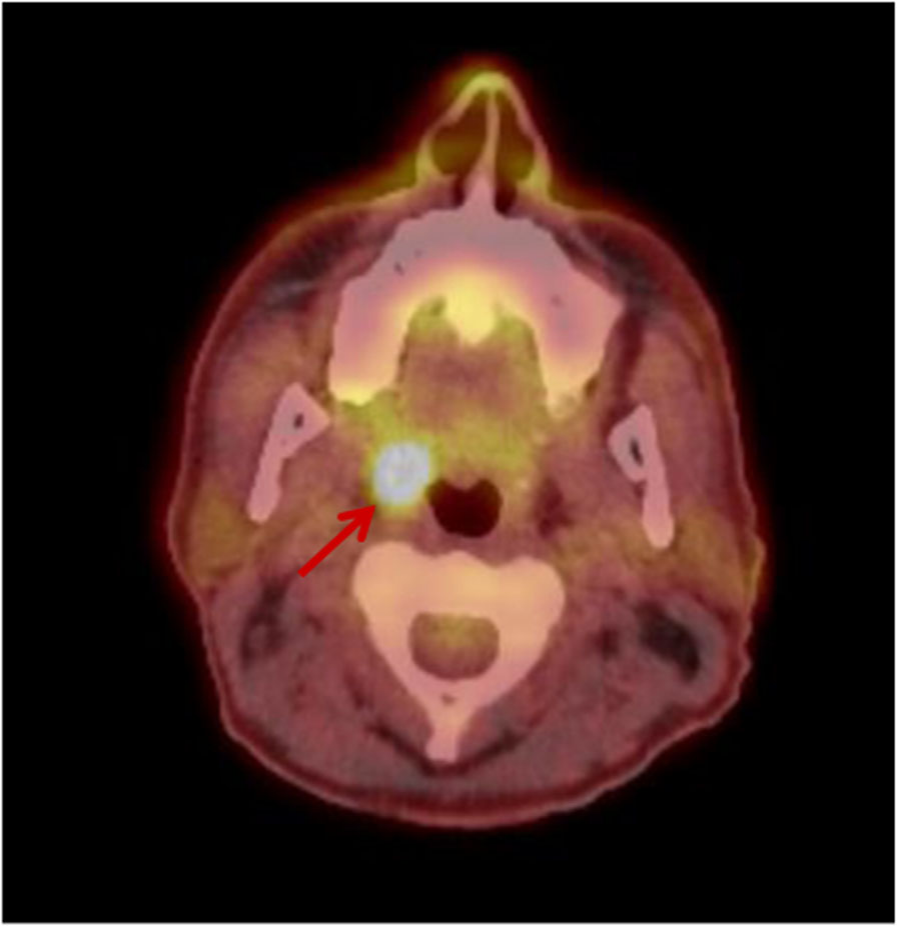
Axial PET-CT scan. An asymmetric contrast medium enhancement of the *right* tonsil is visible (*arrow*). No enhancement of the *left* tonsil

A 52-year-old, male patient presented with an eight-week history of a right-sided cervical mass. No further complaints were mentioned. The patient did not consume alcohol nor did he smoke. The oropharyngeal and laryngeal examination revealed just a slight enlargement of the right tonsil compared to the left without induration of the tonsils, ulceration or other abnormalities. An ultrasound of the neck revealed a highly suspect lymph node formation on the right side (TNM: cN2a). A lymph node extirpation followed and indicated a lymph node metastasis of a low-grade keratinizing squamous cell carcinoma. At this point, no HPV diagnostic of the lymph node material was performed. A PET-CT scan performed two weeks after revealed a highly increased metabolism of the right tonsil with highly increased contrast medium uptake and the suspected diagnosis of a tonsillar carcinoma of the right side (Fig. 2). A panendoscopy with bilateral tonsillectomy and systematic biopsies -including a bilateral deep biopsy of the base of tongue- was performed as a standard procedure for CUP staging/diagnostics. Intraoperatively, an induration of the right tonsil was palpated. Neither the contralateral side, nor the base of tongue showed any abnormalities. However, the pathohistological examination showed a synchronous *bilateral* T1 tonsil squamous cell carcinoma with a HPV-16 positivity in the DNA-PCR analysis of tumor tissue. The patient underwent a bilateral tumor resection with a modified radical neck dissection, level I-V of the right side and a left-sided selective neck dissection, level II-V. The TNM-stage was bilateral pT1 pN2a cM0. Adjuvant radiotherapy followed in the absence of extracapsular spread.

### Discussion

The presence of a bilateral tonsillar carcinoma is rare and only 29 cases are reported in literature. Although many published articles on this topic exist and there is a general consensus that a panendoscopy with representative biopsies should be performed, diverse opinions persist for the handling of the palatine tonsils. An unanimity exists for ipsilateral tonsillectomy but a bilateral procedure is discussed controversially [1].

Although the morbidity of an extended radiation field due to a bilateral primary is increased, the oncologic outcome of missing the second primary by surgery and radiation might be fatal [22]. Due to our clinical experience and the cases reported in literature we provide evidence for bilateral tonsillectomy in cases of CUP-syndrome and unilateral tonsillar cancer with and without HPV-positivity.

#### A tonsillar carcinoma is more likely to be missed by biopsies than by bilateral tonsillectomy

Simo et al. also described a case of a CUP-syndrome with the detection of primary tumor in a tonsillar remnant by status post tonsillectomy in childhood [23]. Through biopsies, risk of false negative results can arise if the tumor is localized in the submucosa or in deep crypts [24] necessitating another attempt to obtain a representative sample.

The standard therapeutic procedure for unilateral tonsillar carcinoma with T-classification T3 or higher is an adjuvant radiation of the contralateral neck even in an N0 stadium since the risk for contralateral lymph node metastasis is approximately 21% [25]. The contralateral tonsil is usually excluded from the irradiation field to avoid the higher morbidity and oropharyngeal complications. Therefore, a bilateral tonsillectomy seems to be justified. This procedure can prevent the consequences of a late diagnosis as well as improved patient outcome compared to a late diagnosis of metachronous tumor [18]. Another advantage is the resulting symmetric appearance of the palate arches, which allows for improved oncologic surveillance. In this case a recurrence or a secondary tumor can be more easily detected by means of the disturbance of the symmetry.

#### Questions of pathogenesis

It is well known that synchronous or metachronous oropharyngeal carcinomas can occur by field cancerization due to symmetrical exposure to noxa [26]. Additionally, recent reports have revealed that Human papilloma viruses (especially subtype 16) increase the risk for developing tonsillar carcinoma [27]. Furthermore, reports exist with speculations of HPV-related oropharyngeal field cancerization and of HPV-related bilateral tonsillar [28]. As we see in Table 1, 6 publications reveal a HPV-positivity in the detected carcinoma in cases with primary diagnosis of CUP-syndrome as well as unilateral tonsillar carcinoma. As it is visible, HPV testing was performed in the more recent publications, demonstrating the relatively new knowledge about the influence of HPV in oral cancer. We present here another case of HPV-positive bilateral tonsillar carcinoma.

In our case, a HPV examination of the metastatic lymph node was not performed and a PET-CT followed to locate a primary tumor. Because of the fact, that the primary tumor in HPV-positive cancers is often occult, a HPV detection in the metastatic lymph node would be helpful for identifying the primary location since 80–90% of HPV positive tumors can be found in the oropharynx (palatine tonsils, base of tongue, lingual tonsils) [1, 29, 30]. Consequently, a HPV positivity in the resected lymph node could be an additional hint and could facilitate the decision of performing a PET-CT or not. This additional examination is not yet a standardized procedure in our clinic but it is in the process of establishment.

Another relevant question in this context would be if it is justified to perform an additional ablation of the base of tongue in HPV-related CUP to minimize the risk of overseeing the primary tumor. Since risk of complications is higher after this procedure and primary tumors are found more common in the palatine tonsils, literature suggests a bilateral base of tongue resection if the palatine tonsils have already been removed [31]. Hence, an ablation of the base of tongue should be discussed if the case of a HPV positive CUP with bilateral tumor-negative tonsils occurs, which would increase the risk of tumor-localization in the base of tongue. This further option has to be taken in consideration and the risk-benefit ratio has to be discussed with the patient since the procedure is painful and is accompanied with increased bleeding risk.

However, a bilateral tonsillectomy with pathological examination of the whole tissue, like in our case performed, is an important procedure in diagnosis and therapeutic management of HPV positive *and* negative tumors, since tonsillar carcinoma often appear in an early stage with manifestation in the deep crypts, not only in HPV positive but also in HPV negative tumors [4].

#### Questions of diagnosis

In our case, PET-CT imaging raised the question of an ipsilateral tonsillar carcinoma as a primary tumor for the existing lymph node metastasis. After bilateral tonsillectomy, a synchronous bilateral carcinoma was histologically detected. A unilateral tonsillectomy would have overlooked the contralateral lesion resulting in a late diagnosis of the contralateral tonsillar carcinoma. PET-CT imaging has proved to be useful in detecting primary sites and distant metastasis in patients with solitary lymph node metastasis. Success rates are reported between 25 and 73%, but a false positive rate is stated between 20 and 46%, which could be explained though increased FDG uptake by chronically inflamed tissue or reactive lymph nodes [32]. False negative results can be caused by early lesions or carcinoma in situ [33]. However, the higher sensitivity of PET-CT makes it more useful for finding the primary tumor site than either PET or CT alone. Nevertheless, in our case described above no reliance on the imaging technique was given. In conclusion, an imaging technique, particularly PET-CT, is necessary for reasons described above and can assist in work-up and diagnosis of CUP-syndrome [18], but a bilateral tonsillectomy with histopathological tissue examination, is more reliable for detection of a tonsillar primary [3] and should not be replaced.

#### Questions of therapy

Bilateral tonsillectomy is recommended as a routine diagnostic tool in 10% of the relevant publications (Table 1). The NCCN 2014 guidelines recommend a tonsillectomy without specifying unilateral or bilateral. Support is growing for the recommendation of bilateral tonsillectomy, but reports can be found with a restrained opinion [1, 24]. For example, Kothari et al. recommend a bilateral tonsillectomy in CUP patients if the PET-CT scan does not reveal any primary [4]. However, this practice can be risky if a false positive result occurs during PET-CT diagnosis, like in our case described. The recommendation of a bilateral tonsillectomy is supported by Moualed et al. who describe two cases of bilateral tonsillar carcinoma with a primary diagnosis of CUP-syndrome and one of bilateral tonsillar carcinoma with a primary diagnosis of being unilateral. They recommend a bilateral tonsillectomy in patients with suspected unilateral tonsillar carcinoma as well as in patients with a cancer of unknown primary [8]. In our clinic we perfom, and therefore we suggest, a bilateral tonsillectomy in non-CUP-cases with a single sided tonsillar carcinoma as well as in all CUP cases regardless HPV status.

An investigation recently published through Fakhry et al. shows a retrospective analysis of the incidence of oropharyngeal cancer after tonsillectomy. The Danish Cancer Registry was analyzed to determine if previous tonsillectomy reduces the future risk for oropharyngeal cancer. They report that remotely performed tonsillectomy resulted in a decreased risk of developing tonsillar cancer [34]. Nevertheless, a prophylactic tonsillectomy can not be recommended and more biomarkers must be developed for the identification of high-risk-persons [35], even more since *Zevallos* et al. demonstrated an increased risk for base of tongue cancer after previously performed tonsillectomy [36].

Arguments against the contralateral tonsillectomy include the potentially increased morbidity associated with rare but severe complications of a post-tonsillectomy bleeding [24]. The new German guidelines recommend a *unilateral* tonsillectomy in the case of a unilateral peritonsillar abscess except for patients with a positive history of recurrent acute tonsillitis, where a bilateral tonsillectomy could be justified. However, the morbidity of a bilateral tonsillectomy does not seem to be significantly greater [3, 10]. Anatomical reasons can be the normal architecture of the contralateral tonsil, compared to the increased vascularization of a pathologically changed tonsil. Nevertheless, clinical trials are necessary with primary endpoint the bleeding risk for reaching representative results. However, extensive tonsillectomy in the context of a very progressive unilateral tonsillar carcinoma can be the cause of an impaired blood supply of the palate and palatal arch with severe consequences in case of a planned reconstruction with a free lap (a.e. m. radialis-transplantation). If a reconstructive procedure is foreseen, this complication has to be considered before a bilateral tonsillectomy is fulfilled.

#### The patient’s perspective

Regarding all steps of diagnosis and therapy, the patient’s point of view is not highlighted. After diagnosis, a fast procedure through the above-mentioned examinations is of great importance as the patient’s focus is in first line a treatment of the disease in a timely manner. Whether a surgical treatment or a therapy through radiation and chemotherapy is needed, depends in most cases on the TNM stadium. In the case that tumor tonsillectomy with or without neck dissection presents the best option, an additional tonsillectomy of the other side with eventually further reconstructions might be a further stress factor for the patient and accompanied with higher risk of complications like dehiscence or necrosis of the transplanted lap, difficulties in swallowing and in food intake. Nevertheless, if this procedure presents the best option to cure the current cancer disease and to prevent/decrease the risk of secondary carcinomas of the opposite side, which will be discussed prior to intervention with the patient, the proposed procedure will be easier accepted.

## Conclusion

It is possible that metachronous tumors of the contralateral tonsil are actually synchronous bilateral tonsil carcinomas, which had not originally been diagnosed. A bilateral tonsillectomy as a diagnostic and partial therapeutic procedure in patients with diagnosis of CUP or confirmed unilateral tonsillar carcinoma should be established as a standard procedure regardless HPV-status. In doing so, the therapy and prognosis can be crucially influenced and the risk for secondary metachronous tumors of the contralateral side can be reduced. The examination of the lymph node metastasis to HPV positivity can give additional hints to an oropharyngeal origin of the primary tumor and should therefore be established in the cascade of CUP-diagnosis. A PET-CT imaging should serve as an indicative investigation and as a supportive diagnostic procedure, but it should not displace panendoscopy with methodical biopsies in combination with a bilateral tonsillectomy. In cases of further soft tissue reconstructions, the possible complication of an alteration of the palatal vasculature has to be considered and an individualized plan needs to be justified.

## Abbreviations

CUP: Cancer of unknown primary
FDG: Fluordesoxyglucose
H&N: Head and neck
HNSCC: Head and neck squamous cell carcinoma
PET-CT: Positron Emission Tomography – Computed Tomography

## References

[1] Cianchetti M, Mancuso AA, Amdur RJ, Werning JW, Kirwan J, Morris CG, Mendenhall WM (2009). Diagnostic evaluation of squamous cell carcinoma metastatic to cervical lymph nodes from an unknown head and neck primary site. Laryngoscope.

[2] Lanzer M, Bachna-Rotter S, Graupp M, Bredell M, Rucker M, Huber G, Reinisch S, Schumann P (2015). Unknown primary of the head and neck: A long-term follow-up. J Craniomaxillofac Surg.

[3] Moualed D, Qayyum A, Price T, Sharma A, Mahendran S (2012). Bilateral synchronous tonsillar carcinoma: a case series and review of the literature. Eur Arch Otorhinolaryngol.

[4] Kothari P, Randhawa PS, Farrell R (2008). Role of tonsillectomy in the search for a squamous cell carcinoma from an unknown primary in the head and neck. Br J Oral Maxillofac Surg.

[5] Jones AS, Morar P, Phillips DE, Field JK, Husband D, Helliwell TR (1995). Second primary tumors in patients with head and neck squamous cell carcinoma. Cancer.

[6] Schwartz LH, Ozsahin M, Zhang GN, Touboul E, De Vataire F, Andolenko P, Lacau-Saint-Guily J, Laugier A, Schlienger M (1994). Synchronous and metachronous head and neck carcinomas. Cancer.

[7] Joseph AW, Ogawa T, Bishop JA, Lyford-Pike S, Chang X, Phelps TH, Westra WH, Pai SI (2013). Molecular etiology of second primary tumors in contralateral tonsils of human papillomavirus-associated index tonsillar carcinomas. Oral Oncol.

[8] Schondorf J, Scherer J (1971). Bilateral tonsillar carcinoma. HNO.

[9] Mannina EM, Pejavar SM, Glastonbury CM, van Zante A, Wang SJ, Yom SS (2011). Diagnosis of Bilateral Tonsil Cancers via Staging PET/CT: Case Report and Review. Int J Otolaryngol.

[10] Koch WM, Bhatti N, Williams MF, Eisele DW (2001). Oncologic rationale for bilateral tonsillectomy in head and neck squamous cell carcinoma of unknown primary source. Otolaryngol Head Neck Surg.

[11] Patel AB, Hinni ML, Pollei TR, Hayden RE, Moore EJ. Severe prolonged dysphagia following transoral resection of bilateral synchronous tonsillar carcinoma. Eur Arch Otorhinolaryngol. 2015; doi:10.1007/s00405-015-3540-x. 10.1007/s00405-015-3540-x 25663269

[12] Bakkal BH, Ugur MB, Bahadir B (2014). Bilateral synchronous squamous cell tonsil carcinoma treated with chemoradiotherapy. JPMA J Pak Med Assoc.

[13] Nakahara S, Yasui T, Takenaka Y, Yamamoto Y, Yoshii T, Morii E, Inohara H (2014). Synchronous bilateral tonsillar carcinomas associated with human papillomavirus. Auris Nasus Larynx.

[14] Smith RO, Pokala K, Medina JE, Krempl GA (2010). Tonsillar carcinoma in the contralateral tonsil. Laryngoscope.

[15] Monsted JE (2010). Bilateral squamous cell carcinoma of the tonsils. Ugeskr Laeger.

[16] Roeser MM, Alon EE, Olsen KD, Moore EJ, Manduch M, Wismayer DJ (2010). Synchronous bilateral tonsil squamous cell carcinoma. Laryngoscope.

[17] Kozakiewicz J, Dec M, Miszczyk L, Urbanczyk H (2007). The rare case of simultaneous bilateral cancer of tonsilla palatina with large metastases to lymphoid glands of the neck. Otolaryngol Pol.

[18] Kazak I, Haisch A, Jovanovic S (2003). Bilateral synchronous tonsillar carcinoma in cervical cancer of unknown primary site (CUPS). Eur Arch Otorhinolaryngol.

[19] Rajenderkumar D, Chan KK, Hayward KA, McRae RD (1999). Bilateral synchronous tonsillar carcinoma. J Laryngol Otol.

[20] Pajor A, Niebudek-Bogusz E, Kaczmarczyk D (1995). Second primary malignant neoplasms in patients treated in the Otolaryngology Clinic AM of Lodz in the years 1981–1989. Otolaryngol Pol.

[21] Price T, Pickles J (2006). Synchronous bilateral tonsillar carcinoma: role of fluoro-deoxyglucose positron emission tomography scanning in detecting occult primary tumours in metastatic nodal disease of the head and neck. J Laryngol Otol.

[22] Reddy AN, Eisele DW, Forastiere AA, Lee DJ, Westra WH, Califano JA (2005). Neck dissection followed by radiotherapy or chemoradiotherapy for small primary oropharynx carcinoma with cervical metastasis. Laryngoscope.

[23] Simo R, O’Connell M (2008). Metastatic squamous cell carcinoma of occult primary: beware the tonsillar remnant. J Laryngol Otol.

[24] Tanzler ED, Amdur RJ, Morris CG, Werning JW, Mendenhall WM. Challenging the need for random directed biopsies of the nasopharynx, pyriform sinus, and contralateral tonsil in the work up of unknown primary squamous cell carcinoma of the head and neck. Head Neck. 2014; doi:10.1002/hed.23931. 10.1002/hed.23931 25488125

[25] Lim YC, Lee SY, Lim JY, Shin HA, Lee JS, Koo BS, Kim SH, Choi EC (2005). Management of contralateral N0 neck in tonsillar squamous cell carcinoma. Laryngoscope.

[26] Slaughter DP, Southwick HW, Smejkal W (1953). Field cancerization in oral stratified squamous epithelium; clinical implications of multicentric origin. Cancer.

[27] D’Souza G, Kreimer AR, Viscidi R, Pawlita M, Fakhry C, Koch WM, Westra WH, Gillison ML (2007). Case-control study of human papillomavirus and oropharyngeal cancer. N Engl J Med.

[28] Chepeha D, Eisbruch A (2010). Commentary: clinical nodal staging of human papillomavirus-related oropharyngeal cancer. Cancer J (Sudbury, Mass).

[29] Begum S, Gillison ML, Ansari-Lari MA, Shah K, Westra WH (2003). Detection of human papillomavirus in cervical lymph nodes: a highly effective strategy for localizing site of tumor origin. Clin Cancer Res.

[30] Chernock RD, Lewis JS (2015). Approach to metastatic carcinoma of unknown primary in the head and neck: squamous cell carcinoma and beyond. Head Neck Pathol.

[31] Byrd JK, Smith KJ, de Almeida JR, Albergotti WG, Davis KS, Kim SW, Johnson JT, Ferris RL, Duvvuri U (2014). Transoral Robotic Surgery and the Unknown Primary: A Cost-Effectiveness Analysis. Otolaryngol Head Neck Surg.

[32] Calabrese L, Jereczek-Fossa BA, Jassem J, Rocca A, Bruschini R, Orecchia R, Chiesa F (2005). Diagnosis and management of neck metastases from an unknown primary. Acta Otorhinolaryngolog Ital.

[33] Shintani SA, Foote RL, Lowe VJ, Brown PD, Garces YI, Kasperbauer JL (2008). Utility of PET/CT imaging performed early after surgical resection in the adjuvant treatment planning for head and neck cancer. Int J Radiat Oncol Biol Phys.

[34] Fakhry C, Andersen KK, Christensen J, Agrawal N, Eisele DW (2015). The Impact of Tonsillectomy upon the Risk of Oropharyngeal Carcinoma Diagnosis and Prognosis in the Danish Cancer Registry. Cancer Prev Res (Philadelphia, Pa).

[35] Misiukiewicz K, Posner M (2015). Role of Prophylactic Bilateral Tonsillectomy as a Cancer Preventive Strategy. Cancer Prev Res (Philadelphia, Pa).

[36] Zevallos JP, Mazul AL, Rodriguez N, Weissler MC, Brennan P, Anantharaman D, Abedi-Ardekani B, Neil Hayes D, Olshan AF (2016). Previous tonsillectomy modifies odds of tonsil and base of tongue cancer. Br J Cancer.

